# Validity, reliability, and comparison of the Indonesian version of two baumann skin type indicator (BSTI) questionnaires

**DOI:** 10.1371/journal.pone.0343028

**Published:** 2026-04-02

**Authors:** Irwan Saputra Batubara, Irma Bernadette S. Sitohang, Sandra Widaty, Hanny Nilasari, Aria Kekalih, Sari Chairunnisa

**Affiliations:** 1 Department of Dermatology and Venereology, Faculty of Medicine, Universitas Indonesia, Dr. Cipto Mangunkusumo Hospital – Jakarta, Indonesia; 2 Dr. Cipto Mangunkusumo Hospital, Jakarta, Indonesia; 3 Medical Staff Unit of Dermatology and Venereology, Faculty of Medicine, Universitas Indonesia, Universitas Indonesia Hospital – Depok, Indonesia; 4 Department of Community Medicine, Faculty of Medicine, Universitas Indonesia, Indonesia; 5 Paragon Technology and Innovation, Ltd., Jakarta, Indonesia; Airlangga University Faculty of Medicine: Universitas Airlangga Fakultas Kedokteran, INDONESIA

## Abstract

**Background:**

Accurate skin type classification is essential for dermatological care and research; however, commonly used classification systems do not adequately capture the multidimensional nature of skin characteristics. The Baumann Skin Type Indicator (BSTI) was developed as a questionnaire-based tool to address this limitation, and a simplified version was introduced in 2022 to improve clinical usability. To date, no validated multidimensional skin type assessment tool is available for the Indonesian population.

**Objectives:**

To evaluate the validity and reliability of the Indonesian translations of the 2006 and 2022 versions of the Baumann Skin Type Indicator (BSTI-Ina) and to assess the level of agreement between the two versions when applied to Indonesian adults.

**Methods:**

A cross-sectional study was conducted using Bahasa Indonesia versions of the 2006 and 2022 BSTI questionnaires, which were distributed online to 150 adult respondents. Content and construct validity were assessed, internal consistency was evaluated using Cronbach’s alpha, and agreement between the two versions was analyzed using the intraclass correlation coefficient (ICC).

**Results:**

Construct validity coefficients ranged from –0.037 to 0.751 for the 2006 BSTI-Ina and from 0.231 to 0.685 for the 2022 version. Cronbach’s alpha values were ranged from 0.613–0.718 for the 2006 version and were 0.704 for the 2022 version, indicating acceptable internal consistency. The ICC between twoversions was 0.435, indicating moderate agreement.

**Conclusion:**

Both the 2006 and 2022 Indonesian versions of the BSTI demonstrate acceptable validity and reliability for skin-type classification in Indonesian adults. The shorter 2022 version may offer a more practical option for routine and research use.

## Introduction

Accurate skin type classification is essential for guiding dermatological management, skincare selection, and research outcomes. Skin characteristics are influenced by intrinsic aging and cumulative exposure to environmental factors, including irritants and ultraviolet (UV) radiation, resulting in substantial interindividual variability [1]. Despite this complexity, skin type assessment often relies on simplified classification systems that do not capture multiple relevant skin parameters.

Traditional skin classification systems introduced by Helena Rubinstein in 1910, and categorize skin as dry, oily, combination, or sensitive. Although widely used, this approach does not incorporate pigmentation status, photoaging, or age-related structural changes, limiting its applicability in clinical decision-making and research contexts [2]. These limitations highlight the need for multidimensional tools capable of providing a more comprehensive assessment of skin characteristics.

The Baumann Skin Type Indicator (BSTI), introduced in 2006, was developed to address this need. The BSTI is a questionnaire-based instrument that classifies skin according to four dichotomous parameters—dry or oily, sensitive or resistant, pigmented or non-pigmented, and tight or wrinkled—yielding 16 distinct skin types. Importantly, BSTI classifications are dynamic and may change with environmental exposure, lifestyle factors, hormonal status, medication use, and seasonal or geographic variation, necessitating periodic reassessment [3]. The BSTI has been applied in several populations, including those in the United States, South Korea, and China, with studies demonstrating associations between BSTI-defined skin types and demographic or behavioural factors such as age, smoking, sun exposure, and comorbidities [1, 4, 5].

To improve feasibility in clinical and research settings, a simplified seven-item version of the BSTI was introduced in 2022. This revised version retains the original classification framework while providing a more detailed subdivision of sensitive skin into five categories, allowing for more specific characterization of sensitive skin phenotypes.

Despite the availability of these instruments, no validated multidimensional skin type assessment tool is currently available for the Indonesian population. In Indonesia, the Fitzpatrick skin phototype system is most commonly used, with phototypes III–V predominating in Southeast Asian populations [6, 7]. However, this system primarily reflects UV response and pigmentation and does not encompass other clinically relevant skin characteristics. Furthermore, susceptibility to UV-induced skin damage has been reported across all Fitzpatrick skin types, including types V and VI [8], underscoring the limitations of relying solely on phototype classification.

Given this gap, validation of a comprehensive, questionnaire-based skin classification tool in the Indonesian context is needed. The objective of this study was to evaluate the validity and reliability of the Indonesian translations of the 2006 and 2022 versions of the Baumann Skin Type Indicator (BSTI-Ina) and to assess the level of agreement between the two versions when applied to Indonesian adults.

## Materials and methods

### Study design

This cross-sectional study was conducted in accordance with STROBE guidelines to evaluate the validity and reliability of the 2006 and 2022 Baumann Skin Type Indicator Indonesian versions (BSTI-Ina). The study took place from July to October 2023 at the Department of Dermatology and Venereology, Faculty of Medicine, Universitas Indonesia. Questionnaire distribution and data collection were conducted from 8–22 September 2023. Participants were recruited consecutively.

### Participants

Eligible participants were adults aged 18–59 years with Fitzpatrick skin types III–V, fluent in Bahasa Indonesia, literate, and with a minimum of senior high school education. Exclusion criteria included facial dermatoses involving more than 25% of the face, receipt of cosmetic dermatologic procedures (chemical peels, laser therapy, dermabrasion, botulinum toxin, fillers, or skin-rejuvenation injections) within the previous two weeks, or incomplete responses to either questionnaires.

Based on cross-cultural adaptation guidelines by Sousa et al. (2010), 30 subjects were included in the pre-final phase. A total of 150 participants were recruited for the final validity and reliability assessment [9].

### Ethical Approval

Ethical approval was granted by the Research Ethics Committee, Faculty of Medicine, Universitas Indonesia (KET-174/UN2.F1/ETIK/PPM.00.02/2023). All participants provided written informed consent, with assurances of privacy and confidentiality.

### Translation and cross-cultural adaptation

Permission to translate the questionnaires was obtained from Dr. Leslie Baumann, the original author. Two certified translators, one with a medical background and one without, independently translated the 2006 and 2022 BSTI questionnaires into Bahasa Indonesia. A multidisciplinary committee comprising two dermatologists, a biostatistician, a clinical psychologist, and both translators then reviewed each version to ensure linguistic accuracy, clarity, and cultural appropriateness.

Two additional certified translators, blinded to the original questionnaire, performed back-translation. Forward and backward translations were submitted to Dr. Baumann for review and approval. The back translation of the 2006 and 2022 BSTI questionnaires can be found in S1 and S2 Table.

### Pre-Final Testing

The approved translations underwent pre-final testing among 30 participants using Google Forms. Subjects provided feedback regarding clarity, difficulty, and usability. Suggested modifications were incorporated to refine the questionnaires before final testing.

### Validity and Reliability Assessment

A total of 150 participants completed both BSTI-Ina versions. Content validity was established through expert review and pre-final testing. Construct validity was assessed using Pearson correlations between item scores and total scores with score >0.2 were considered valid [10].

Reliability was assessed using Cronbach’s alpha (α), with α = 0.6–0.8 considered acceptable, α > 0.8 good, and α > 0.9 very good. Statistical analyses were performed using SPSS version 25 [11].

### Correlation between BSTI-Ina versions

Agreement between 2006 and 2022 versions was assessed using the intra-class correlation coefficient (ICC). A minimum ICC threshold of >0.25 was predetermined due to the absence of prior comparative studies between these two instruments.

## Results

### Subject characteristics

The median age of participants was 35 years (IQR 29–44). Most subjects held a bachelor’s degree (50%), while 29.3% had completed high school. Baseline characteristics are presented in Table 1.

**Table 1:** Characteristics of subjects.

Characteristics	Total
Age (year), Median (IQR)	35 (29–44)
Gender, n (%)	
Men	41 (27.3)
Women	109 (72.9)
Marital Status, n (%)	
Not Married	46 (30.7)
Married	100 (66.7)
Divorced/Widow	4 (2.7)
Education, n (%)	
No School	0 (0.0)
Elementary School	0 (0.0)
Junior High School	0 (2.0)
High School	38 (29.3)
Diploma	31 (20.7)
Bachelor	75 (50.0)
Occupation, n (%)	
Housewife	7 (4.7)
Private Sector Employee	48 (32.0)
Civil Servant/TNI/POLRI	45 (30.0)
Self-Employee	9 (6.0)
Student	19 (12.7)
Health-care Worker	21 (14.0)
Others	1 (0.7)

### Pre-final Study

The pre-final questionnaires were tested in 30 native Indonesian speakers. No comprehension issues were reported, and no revisions were required.

### Validity and Reliability Study

Because no modifications were needed, the 30 pre-final participants were included in the final analysis. An additional 120 participants were recruited, yielding a total of 150 participants. Construct validity results for the 2006 BSTI-Ina are shown in Table 2. Reliability was evaluated using Cronbach’s α. For the 2006 BSTI-Ina, α scores were 0.704 (dry/oily), 0.718 (sensitive/resistant), 0.691 (pigmented/non-pigmented), and 0.613 (tight/wrinkled), as shown in Table 3. The 2022 BSTI-Ina demonstrated a Cronbach’s α of 0.704, with corresponding validity and reliability results presented in Table 4.

**Table 2:** Validity of the 2006 BSTI-Ina.

Question	Corrected Item total correlation			
	Dry vs Oily	Sensitive vs Resistant	Pigmented vs Non- pigmented	Tight vs Wrinkle
1	0.585	0.492	0.319	0.093*
2	0.490	0.550	0.390	0.364
3	0.477	0.499	0.347	0.306
4	0.561	0.360	0.660	0.386
5	0.257	0.405	0.630	0.395
6	0.739	0.394	0.440	0.336
7	0.162*	0.145*	0.544	0.266
8	0.298	0.588	−0.002*	0.285
9	0.307	0.271	0.095*	0.249
10	0.621	0.550	0.551	0.172*
11	0.511	0.428	0.546	0.272
12		0.597	−0.037*	0.504
13		0.354	0.279	0.228
14		0.478	0.751	0.339
15		0.475		0.280
16		0.497		0.223
17		0.391		0.392
18		0.562		0.245
19				0.148*
20				0.163*
21				0.323

Note:

Each score was measured from item-to-total correlations within each respective parameter (dry/oily, sensitive/resistant, pigmented/non-pigmented, tight/wrinkled).

*Correlation value with a score less than 0.2

**Table 3:** Reliability test of the 2006 BSTI-Ina.

Sub-Domain of 2006 BSTI-Ina	Cronbach α
Parameter of dry vs oily	0.704*
Parameter of sensitive vs resistant	0.718*
Parameter of pigmented vs non-pigmented	0.691*
Parameter of tight vs wrinkle	0.613*

Note:

*Score of Cronbach α > 0.6

**Table 4:** Validity and reliability test of the 2022 BSTI-Ina.

Sub-Domain of 2022 BSTI-Ina	*Corrected Item total correlation*	Cronbach α
Parameter of dry vs oily	0.685	Cronbach α score for all 2022 BSTI-Ina questions: 0.704*
Parameter of sensitive vs resistant	0.614	
Parameter of pigmented vs non-pigmented	0.669	
Parameter of tight vs wrinkle	0.231	

Note:

*Score of Cronbach α > 0.6

### Correlation between the 2006 and 2022 BSTI-Ina questionnaires

The ICC between the 2006 and 2022 BSTI-Ina was 0.435 (95% CI: 0.245–0.604; p = 0.0001). Skin-type distributions for both versions are presented in Table 5.

**Table 5:** Skin Type of Subjects Based on the 2006 and 2022 BSTI-Ina.

	Total		Men		Women	
Skin Type	2006 BSTI N (%)	2022 BSTI N (%)	2006 BSTI N (%)	2022 BSTI N (%)	2006 BSTI N (%)	2022 BSTI N (%)
DRNT	16 (10.7)	1 (0.7)	6 (4)	1 (0.7)	10 (6.7)	0 (0)
DRNW	7 (4.7)	5 (3.3)	5 (3.3)	1 (0.7)	2 (1.3)	4 (2.7)
DRPT	1 (0.7)	0 (0)	0 (0)	0 (0)	1 (0.7)	0 (0.0)
DRPW	5 (3.3)	6 (4)	1(0.7)	1 (0.7)	4 (2.7)	5 (3.3)
DSNT	10 (6.7)	0 (0)	0 (0)	0 (0)	10 (6.7)	0 (0.0)
DSNW	15 (10.0)	17 (11.3)	4 (2.7)	4 (2.7)	11 (7.3)	13 (8.7)
DSPT	9 (6.0)	3 (2)	0 (0)	0 (0)	9 (6)	3 (2.2)
DSPW	11(7.3)	37 (24.7)	3 (2)	3 (2)	8 (5.3)	34 (22.7)
ORNT	19 (12.7)	1 (0.7)	5 (3.3)	1 (0.7)	14 (9.3)	0 (0.0)
ORNW	8 (5.3)	16 (10.7)	6 (4)	10 (6.7)	2 (1.3)	6 (4.0)
ORPT	4 (2.7)	0 (0)	1 (0.7)	0 (0)	3 (2)	0 (0.0)
ORPW	4 (2.7)	11 (7.3)	2 (1.3)	2 (1.3)	2 (1.3)	9 (6.0)
OSNT	12 (8)	2 (1.3)	0 (0)	2 (1.3)	12 (8)	0 (0.0)
OSNW	12 (8)	24 (16)	6 (4)	12 (8)	6 (4)	12 (8.0)
OSPT	6 (4)	2 (1.3)	0 (0)	1 (0.7)	6 (4)	1 (0.7)
OSPW	11 (7.3)	25 (16.7)	2 (1.3)	3 (2)	9 (6)	22 (14.7)

Note: D: dry, O: oily, R: resistant, S: sensitive, P: pigmented, N: non-pigmented, T: tight, W: wrinkle.

## Discussion

This study demonstrated that both the 2006 and 2022 BSTI-Ina questionnaires are valid and reliable instruments for classifying skin types in Indonesian adults. The translation and cross-cultural adaptation process, conducted by expert committee, ensured linguistic accuracy and conceptual clarity suitable for the Indonesian population.

The pre-final testing phase with 30 native speakers showed that both questionnaires were easy to understand, consistent with recommended guidelines for cross-cultural adaptation [9]. No modifications were required, allowing these participants to be included in the final analysis.

Validity and reliability testing in 150 subjects confirmed strong psychometric performance. Content validity was supported by expert review and participant comprehension. Construct validity results for the 2006 BSTI-Ina showed acceptable correlation coefficients for most items. Several low-scoring items can be explained by population characteristics and response homogeneity.

For the *dry/oily* parameter, correlations ranged from 0.162–0.739. Question 7 (“When you use soap that foams, bubbles, and foams a lot, your facial skin: …”) scored <0.2, possibly due to variations in soap formulations.

For the *sensitive/resistant* parameter (0.145–0.647), Question 7 (“How often do you experience rashes under the ring? …”) scored <0.2 because most participants had never experienced ring rash, likely related to variations in ring materials, with nickel more commonly causing allergic contact dermatitis [12, 13].

For the *pigmented/non-pigmented* parameter (–0.037–0.751), Questions 8 and 9 (“When exposed to sun for the first time in several months, your skin: …”; “What happens after you are exposed to sunlight consecutively for days? …”) scored <0.2, reflecting typical Fitzpatrick IV–V responses, which tan easily and burn less [6]. Question 12 (“What is your natural hair color? …”) showed homogeneous responses, as most subjects selected “black.”

For the *tight/wrinkled* parameter (0.093–0.504), Questions 1, 10, 19, and 20 scored <0.2. Question 1 (“Are there any wrinkles on your face? …”) reflected the median age of 35, corresponding to Glogau II wrinkle patterns.[8] Question 10 (“Based on the places you have lived, how much daily sun exposure have you received…”) also showed low variation because all subjects are reside in Jakarta and its surrounding areas, where sun exposure is consistently high. Questions 19 and 20 (“What is your natural skin color…?” and “What is your ethnicity…?”) showed low variability due to the sample’s homogeneous Indonesian Asian background.

In contrast, all items in the 2022 BSTI-Ina showed correlation coefficients >0.2 (0.231–0.685), confirming its simpler structure and greater item relevance.

Reliability testing demonstrated good internal consistency for both questionnaires. The 2006 BSTI-Ina yielded Cronbach’s α values of 0.704 (dry/oily), 0.718 (sensitive/resistant), 0.691 (pigmented/non-pigmented), and 0.613 (tight/wrinkled), consistent with international studies validating the original 2006 BSTI [4]. The 2022 BSTI-Ina showed a Cronbach’s α of 0.704, indicating good reliability. No prior published studies have evaluated the 2022 BSTI, making this study the first to do so.

Because of the structural differences between these two versions, in which the 2006 BSTI contains 64 questions across four domains, and the 2022 BSTI is a simplified 7-item tool that incorporates a different approach to categorizing wrinkled (W) skin types, including automatic classification for individuals aged ≥30 years, perfect agreement between versions was not expected. Instead, the study aimed to determine whether the shorter 2022 version remains valid and reliable on its own. The ICC between the 2006 and 2022 BSTI-Ina was 0.435, exceeding the predetermined threshold of 0.25 and demonstrating moderate correlation between the two tools. This confirms that, although shorter, the 2022 version maintains conceptual alignment with the 2006 version.

The 2006 BSTI-Ina contains 64 multiple-choice questions across four domains (11 dry/oily, 18 sensitive/resistant, 14 pigmented/non-pigmented, 21 tight/wrinkled), all of which were well understood by participants. All the instructions and questions on the 2006 BSTI-Ina questionnaire are understandable to the subjects.

The 2022 BSTI-Ina simplifies assessment into seven questions. Notably, questions 1 and 2 gather essential personal information, including gender and age. Distinguishing itself from its predecessor, the 2022 version directly classifies individuals aged 30 or older as having a wrinkled (W) skin type. Questions 3 and 4 aim to assess dry or oily skin. Question number 5 addresses the sensitive or resistant skin type, with a notable enhancement in BSTI-Ina 2022. Sensitive skin types are now subcategorized into five specific subtypes: acne, redness or flushing, burning or stinging sensation, allergic reactions, and irritation. Question number 6 focuses on evaluating pigmented or non- pigmented skin. Question number 7 aims to assess wrinkled or tight skin. Wrinkled skin type is assigned if they choose three or more answer choices (excluding “not all”) or if they are 30 years or older. All items were understood clearly, and the shorter format increases practicality for clinical use.

A limitation of this study is the lack of cultural validity testing. Indonesia’s diverse ethnic groups may differ in perceptions of skin type, yet the sample was limited to Jakarta.

A strength of this study is that it is the first to evaluate the validity and reliability of both the 2006 and 2022 BSTI-Ina versions, as well as the first to assess their correlation.

## Conclusion

Both the 2006 and 2022 BSTI-Ina questionnaires were successfully translated, adapted, and validated for use in Indonesian adults. Each demonstrated good validity and reliability, with moderate correlation between versions. The shorter 2022 BSTI-Ina offers a practical and efficient tool for routine skin-type assessment.

## Supporting information

S1 Table Back translation of the 2006 version of BSTI. (DOCX)

S2 Table Back translation of the 2022 version of BSTI. (DOCX)
