# Supplementary material for: Validity, reliability, and comparison of the Indonesian version of two baumann skin type indicator (BSTI) questionnaires
Source: PLoS One. 2026 Apr 2;21(4):e0343028. doi: 10.1371/journal.pone.0343028 (PMC13046154; doi:10.1371/journal.pone.0343028)
Supplement: S1 Table — (DOCX) [file pone.0343028.s001.docx]

**S1 Table. Back translation of the 2006 version of BSTI**

| No | Baumann Skin Type Questionnaire 2006  **(Original)** | Baumann 2006 Skin Type Questionnaire  **(Translator's back translation 1)** | Baumann Skin Type Questionnaire 2006  **(Translator's back translation 2)** |
| --- | --- | --- | --- |
|  | **Part One**  **Oily vs. Dry** | **First Section**  **Oily vs. Dry** | **Part One**  **Oily vs. Dry** |
|  | This section measures skin oil production and hydration. Studies show that peoples’ preconceptions about whether their skin is oily or dry are often inaccurate. Don’t allow your preconceptions or what others think and say about your skin to bias your answers. | This section measures the skin's oil production and hydration. Research shows that people's opinion on condition of their skin (oily or dry) are often inaccurate. Don't let your opinion, or what other people say and think about your skin, influence your answer. | This section measures oil production and skin hydration. Research has shown that people's perceptions of their skin condition (oily or dry) are often inaccurate. Please do not let your own opinions or the opinions of others about your skin influence your answers. |
| 1 | After washing your face, don’t apply any moisturizer, sunscreen, toner, powder, or other products. Two to three hours later, look in a mirror under bright lights. Your forehead and cheeks feel or appear:   1. Very rough, flaky, or ashy 2. Tight 3. Well hydrated with no reflection of light 4. Shiny with reflection of bright light | After washing your face, do not use moisturizer, sunscreen, toner, powder, or other products. Two to three hours later, look in a mirror under a bright light. Your forehead and cheeks feel or look like:   1. Very rough, scaly, or pale 2. Tight 3. Well hydrated with no light reflection 4. Shiny with bright light reflection | After washing your face, do not apply moisturizer, sunscreen, toner, powder, or any other products. Two to three hours later, examine your forehead and cheeks under bright light. Your forehead and cheeks feel or appear:   1. Very rough, flaky, or pale 2. Tight 3. Well-hydrated without any light reflection 4. Shiny with a bright light reflection |
| 2 | In photos, your face appears shiny:   1. Never, or you’ve never noticed shine 2. Sometimes 3. Frequently 4. Always | In the photo, your face looks shiny:   1. Never, or you never notice looks shiny 2. Sometimes 3. Often 4. Always | In photographs, your face looks shiny:   1. Never, or you have never noticed looking shiny 2. Occasionally 3. Often 4. Always |
| 3 | Two to three hours after applying makeup foundation (also known as base) but no powder, your makeup appears:   1. Flaky or caked in wrinkles 2. Smooth 3. Shiny 4. Streaked and shiny 5. I do not wear facial foundation. | Two to three hours after using foundation (which is also known as a makeup base), but not using powder, your makeup looks:   1. Cracks or lumps in the wrinkles 2. Fine 3. shiny 4. Cracked and shiny 5. I don't use foundation. | Two to three hours after using foundation (also known as makeup base) but without using powder, your makeup looks:   1. Cracked or clumped in wrinkles 2. Smooth 3. Shiny 4. Cracked and shiny 5. I don't use foundation. |
| 4 | When in a low-humidity environment, if you don’t use moisturizers or sunscreen, your facial skin:   1. Feels very dry or cracks 2. Feels tight 3. Feels normal 4. Looks shiny, or I never feel that I need moisturizer 5. Don’t know | When in a low humidity environment, if you don't use moisturizer or sunscreen, your facial skin:   1. Feels very dry or cracked 2. Feels tight 3. Feels normal 4. It looks glossy, or I never feel that I need a moisturizer 5. Don't know | When in a low-humidity environment, if you do not use moisturizer or sunscreen, your facial skin feels:   1. Very dry or chapped 2. Tight 3. Normal 4. Shiny, or I never feel the need for moisturizer 5. I don't know. |
| 5 | Look in a magnifying mirror. How many large pores, the size of the end of a pin or greater, do you have?   1. None 2. A few in the T-zone (forehead and nose) only 3. Many 4. Tons! 5. Don’t know (Note: Please look again and only answer *e* if you cannot determine this.) | Look in a magnifying mirror. How many large pores, the size of needle tip or larger, do you have?   1. There isn't any 2. Few, only in the T-zone (forehead and nose) 3. Lots 4. Many! 5. Don't know (Note: Please look again and only answer e if you cannot determine the answer.) | Please examine your face using a magnifying mirror. How many pores on your skin, that are as big as the tip of a needle or larger, do you have?   1. None 2. Few, only in the T-zone (forehead and nose) 3. Many 4. A lot! 5. I don't know (Note: Please look again and only select e if you cannot determine the answer.) |
| 6 | You would characterize your facial skin as:   1. Dry 2. Normal 3. Combination 4. Oily | In your opinion, your facial skin is classified as:   1. Dry 2. Normal 3. Combination 4. Oily | In your opinion, your facial skin is classified as:   1. Dry 2. Normal 3. Combination 4. Oily |
| 7 | When you use soap that suds, bubbles, and foams vigorously, your facial skin:   1. Feels dry or cracks 2. Feels slightly dry but does not crack 3. Feels normal 4. Feels oily 5. I do not use soap or other foaming cleansers. (If this is because they make your skin dry, pick a.) | When you use a soap that lathers, bubbles, and lathers a lot, your facial skin:   1. Feels dry or cracked ‘ 2. Feels a bit dry but not cracked 3. Feels normal 4. Feels greasy 5. I don't use soap or other foaming cleansers. (If it's because the soap is drying your skin, choose a.) | When you use a foaming, bubbly, and highly lathering soap, your facial skin feels:   1. Dry or cracked 2. Somewhat dry but not cracked 3. Normal 4. Oily 5. I don't use soap or other foaming cleansers. (If this is because soap makes your skin dry, select a.) |
| 8 | If not moisturized, your facial skin feels tight:   1. Always 2. Sometimes 3. Rarely 4. Never | If you don't moisturize, your facial skin feels tight:   1. Always 2. Sometimes 3. Seldom 4. Never | If not provided with moisturizer, your facial skin feels tight:   1. Always 2. Sometimes 3. Rarely 4. Never |
| 9 | You have clogged pores (blackheads or whiteheads):   1. Never 2. Rarely 3. Sometimes 4. Always | You have clogged pores (blackheads or whiteheads):   1. Never 2. Seldom 3. Sometimes 4. Always | You have clogged pores (blackheads or whiteheads):   1. Never 2. Rarely 3. Sometimes 4. Always |
| 10 | Your face is oily in the T-zone (forehead and nose):   1. Never 2. Sometimes 3. Frequently 4. Always | Your face is oily in the T-zone (forehead and nose area):   1. Never 2. Sometimes 3. Often 4. Always | Your face is oily in the T-zone (forehead and nose area):   1. Never 2. Occasionally 3. Often 4. Always |
| 11 | Two to three hours after applying moisturizer your cheeks are:   1. Very rough, flaky, or ashy 2. Smooth 3. Slightly shiny 4. Shiny and slick, or I do not use moisturizer | Two to three hours after applying your cheek moisturizer to:   1. Very rough, scaly, or pale 2. Fine 3. A little shiny 4. Shiny and slippery, or I don't use moisturizer pelembab | Two to three hours after applying moisturizer to your cheeks, they become:   1. Very rough, flaky, or pale 2. Smooth 3. Slightly shiny 4. Shiny and smooth, or I don't use moisturizer. |
|  | **Part Two**  **Sensitive vs. Resistant** | **Section Two**  **Sensitive vs. resistance** | **Part Two**  **Sensitive vs. Resistant** |
|  | This section measures your skin’s tendency to develop pimples, redness, flushing, and itching, all signs of sensitive skin. | This section measures your tendency to experience skin breakouts, redness, flushing, and itching; all are signs of sensitive skin. | This section measures your tendency to experience acne, redness, flushing (sudden redness and warmth in the face, neck, and chest), and itching; all of which are signs of sensitive skin. |
| 1 | You get red bumps on your face:   1. Never 2. Rarely 3. At least once a month 4. At least once a week | You have red bumps on your face:   1. Never 2. Seldom 3. At least once a month 4. At least once a week | You have red bumps on your face:   1. Never 2. Rarely 3. At least once a month 4. At least once a week |
| 2 | Skin care products (including cleanser, moisturizer, toners, and makeup) cause your face to break out, get a rash, itch, or sting:   1. Never 2. Rarely 3. Often 4. Always 5. I don’t wear products on my face. | Skin care products (including cleansers, moisturizers, toners, and cosmetics) cause your face to break out, develop a rash, itch, or sting:   1. Never 2. Seldom 3. Often 4. Always 5. I don't use any product on my face. | Skincare products (including cleansers, moisturizers, toners, and cosmetics) cause acne, rashes, itching, or stinging on your face:   1. Never 2. Rarely 3. Often 4. Always 5. I don't use any products on my face. |
| 3 | Have you ever been diagnosed with acne or rosacea?   1. No 2. Friends and acquaintances tell me I have it. 3. Yes 4. Yes, a severe case 5. Unsure | Have you ever been diagnosed with acne or rosacea?   1. No 2. Friends and acquaintances say that I experienced it. 3. Yes 4. Yes, including severe cases 5. Not sure | Have you been diagnosed with acne or rosacea?   1. No 2. Friends and acquaintances have told me I have it. 3. Yes 4. Yes, including severe cases 5. Not sure |
| 4 | If you wear jewelry that is not 14-carat gold, how often do you get a rash?   1. Never 2. Rarely 3. Often 4. Always 5. Unsure | If you wear jewelry that is not gold or less than 14 carat gold, how often do you get a rash?   1. Never 2. Seldom 3. Often 4. Always 5. Not sure | How frequently do you encounter skin irritations when you wear non-gold jewelry or gold that is below 14 karats?   1. Never 2. Rarely 3. Often 4. Always 5. Not sure |
| 5 | Sunscreens make your skin itch, burn, break out, or turn red:   1. Never 2. Rarely 3. Often 4. Always 5. I never wear sunscreen. | Sunscreen makes your skin itch, burn, break out, or blush:   1. Never 2. Seldom 3. Often 4. Always 5. I never wear sunscreen. | Sunscreen makes your skin feel itchy, burning, acne-prone, or red:   1. Never 2. Rarely 3. Often 4. Always 5. I never use sunscreen. |
| 6 | Have you ever been diagnosed with atopic dermatitis, eczema, or contact dermatitis (an allergic skin rash)?   1. No 2. Friends tell me I have it. 3. Yes 4. Yes, a severe case 5. Unsure | Have you ever been diagnosed with atopic dermatitis, eczema, or contact dermatitis (skin rash due to allergies)?   1. No 2. Friends say that I experienced it. 3. Yes 4. Yes, including severe cases 5. Not sure | Have you been diagnosed with atopic dermatitis, eczema, or contact dermatitis (skin rash due to allergies)?   1. No 2. Friends have told me I have it. 3. Yes 4. Yes, including severe cases 5. Not sure |
| 7 | How often do you get a rash underneath your rings?   1. Never 2. Rarely 3. Often 4. Always 5. I do not wear rings. | How often do you get a rash under the ring?   1. Never 2. Seldom 3. Often 4. Always 5. I don't wear rings | How frequently do you suffer from skin irritations beneath your ring?   1. Never 2. Rarely 3. Often 4. Always 5. I don't wear rings. |
| 8 | Fragranced bubble bath, massage oil, or body lotions make your skin break out, itch, or feel dry:   1. Never 2. Rarely 3. Often 4. Always 5. I never use these types of products. (Note: answer d if you don’t use them because they cause the above-mentioned problems.) | Scented products like foaming body wash, massage oil, or lotion make your skin break out, itch, or feel dry:   1. Never 2. Seldom 3. Often 4. Always 5. I have never used such a product. (Note: answer d if you do not use the product because it causes the problem above.) | Scented products such as foaming body wash, massage oil, or lotion result in acne, itching, or dryness on your skin.   1. Never 2. Rarely 3. Often 4. Always 5. I never use such products. (Note: choose d if you don't use these products because they cause the above-mentioned problems.) |
| 9 | Can you use the soap provided in hotels on your body or face without a problem?   1. Yes 2. Most of the time, I don’t have a problem. 3. No, my skin itches, turns red, or breaks out 4. I would not use it. I’ve had too many problems in the past! 5. I carry my own, so I’m unsure. | Can you use the soap provided at the hotel on your body or face without problems?   1. Yes 2. Most of the time, no problem. 3. No, my skin is itchy, red, or has breakouts 4. I won't use it. Too many problems already! 5. I brought my own product, so I'm not sure. | Can you use the soap provided in hotels for your body or face without any issues on your skin?   1. Yes 2. Often, no problems. 3. No, my skin becomes itchy, red, or breaks out. 4. I wouldn't use it. It has caused problems too many times! 5. I bring my own products, so I'm not sure. |
| 10 | Has someone in your family been diagnosed with atopic dermatitis, eczema, asthma, and/or allergies?   1. No 2. One family member that I know of 3. Several family members 4. Many of my family members have dermatitis, eczema, asthma, and/or allergies. 5. Unsure | Has anyone in your family been diagnosed with atopic dermatitis, eczema, asthma, and/or allergies?   1. No 2. There is one family member that I know 3. There are several family members 4. Many members of my family have dermatitis, eczema, asthma and/or allergies. 5. Not sure | Does anyone in your family have a diagnosis of atopic dermatitis, eczema, asthma, and/or allergies?   1. No 2. One family member that I know of. 3. Several family members 4. Many family members have dermatitis, eczema, asthma, and/or allergies. 5. Not sure |
| 11 | What occurs if you use scented laundry detergents or static control sheets in the dryer?   1. My skin is fine. 2. My skin feels slightly dry. 3. My skin itches. 4. My skin itches and gets a rash. 5. Unsure, or I’ve never used them | What happens if you use a laundry detergent that contains fragrance?   1. My skin is fine 2. My skin feels a little dry. 3. My skin itches. 4. My skin itches and has a rash. 5. Not sure, or I never used it | What happens when you use scented laundry detergent?   1. My skin is fine. 2. My skin feels slightly dry. 3. My skin itches. 4. My skin itches and breaks out. 5. Not sure, or I've never used it. |
| 12 | How often do your face and/or neck get red after moderate exercise, and/or with stress or a strong emotion, such as anger?   1. Never 2. Sometimes 3. Frequently 4. Always | How often does your face and/or neck turn red after moderate intensity exercise, and/or when you are stressed or experiencing strong emotions such as anger?   1. Never 2. Sometimes 3. Often 4. Always | How frequently does your face and/or neck become red following moderate physical activity or during times of stress or intense emotions such as anger?   1. Never 2. Sometimes 3. Often 4. Always |
| 13 | How often do you tend to get red and flushed after drinking alcohol?   1. Never 2. Sometimes 3. Frequently 4. Always, or I don’t drink because of this problem 5. I never drink alcohol. | How often do your skin tend to get red and feel hot/hot (flushing) after drinking alcohol?   1. Never 2. Sometimes 3. Often 4. Always, or I don't drink because of this problem 5. I never drink alcohol | How frequently does your skin become red and feel warm/flushed after consuming alcohol?   1. Never 2. Sometimes 3. Often 4. Always, or I don't drink because of this issue 5. I never drink alcohol. |
| 14 | How often do you get red and flushed after eating spicy or hot (temperature) foods or beverages?   1. Never 2. Sometimes 3. Frequently 4. Always 5. I never eat spicy food. (Note: if you don’t eat spicy or hot food because of facial flushing, pick d.) | How often does your skin get red and feel hot/hot (flushing) after consuming hot or spicy drinks or food?   1. Never 2. Sometimes 3. Often 4. Always 5. I never eat spicy food. (Note: if you don't eat spicy or hot food because of flushing, choose d.) | How frequently does your skin turn red and feel warm/flushed after consuming spicy food or hot beverages?   1. Never 2. Occasionally 3. Often 4. Always 5. I never eat spicy food. (Note: if you don't eat spicy or hot food because of flushing, choose d.) |
| 15 | How many visible red or blue broken blood vessels do you have (or did you have prior to treatment) on your face and nose?   1. None 2. Few (one to three on entire face, including nose) 3. Some (four to six on entire face, including nose) 4. Many (over seven on entire face, including nose) | How many red or blue veins are visible on your face and nose now (or before the treatment)?   1. There isn't any 2. A little (one to three all over the face, including the nose) 3. Several (four to six across the face, including the nose) 4. Multiple (more than seven on entire face, including nose) | How many visible red or blue blood vessels do you have on your face and nose currently (or prior to any treatments)?   1. None 2. A few (one to three on the entire face, including the nose) 3. Some (four to six on the entire face, including the nose) 4. Many (more than seven on the entire face, including the nose) |
| 16 | Your face looks red in photographs:   1. Never, or I never noticed it 2. Sometimes 3. Frequently 4. Always | Your face looks red in photos:   1. Never, or I never noticed 2. Sometimes 3. Often 4. Always | Your face looks red in photographs:   1. Never, or I've never noticed it. 2. Occasionally 3. Often 4. Always |
| 17 | People ask you if you are sunburned, even when you are not:   1. Never 2. Sometimes 3. Frequently 4. Always 5. I always am sunburned. (You bad thing!) | People ask if you get a sunburn, even when you don't:   1. Never 2. Sometimes 3. Often 4. Always 5. My skin is always sunburned. (It sucks!) | People ask if your skin is sunburned, even when it's not:   1. Never 2. Occasionally 3. Often 4. Always 5. My skin is always sunburned. (So annoying!) |
| 18 | You get redness, itching, or swelling from makeup, sunscreen, or skin care products:   1. Never 2. Sometimes 3. Frequently 4. Always 5. I do not use these products. (Note: answer d if you don’t use them because of redness, itching, or swelling.)   If you’ve ever received a diagnosis of acne, rosacea, contact dermatitis, or eczema from a dermatologist, add 5 to your score. If another type of physician has diagnosed you with these conditions, add 2 to your score. | You experience redness, itching, or swelling from cosmetics, sunscreen, or skin care products:   1. Never 2. Sometimes 3. Often 4. Always 5. I don't use those products. (Note: answer d if you don't use it because of redness, itching, or swelling.)   If you have been diagnosed with acne, rosacea, contact dermatitis, or eczema by a dermatologist, add 5 points to your score. If diagnosed by a specialist other than skin, add 2 points to your score. | Do you encounter any redness, itching, or swelling as a result of using cosmetics, sunscreen, or skincare products?   1. Never 2. Occasionally 3. Often 4. Always 5. I don't use those products. (Note: choose d if you don't use them because of experiencing redness, itching, or swelling.)   If a dermatologist has diagnosed you with acne, rosacea, contact dermatitis, or eczema, add 5 points to your score. If the diagnosis was made by a specialist other than a dermatologist, add 2 points to your score. |
|  | **Part Three**  **Pigmented vs. Non-Pigmented Skin** | **Section Three**  **Pigmented Skin vs. Not Pigmented** | **Part Three**  **Pigmented vs. Non-Pigmented Skin** |
|  | This section measures your skin’s tendency to form melanin, a skin pigment that produces darker skin tones as well as dark patches, freckles, and dark areas after trauma. Melanin also helps you tan rather than burn. | This section measures your skin's tendency to form melanin; a skin pigment that produces a darker color on the skin, and causes dark spots, freckles, and dark scars. Melanin also helps make the skin darker (tanning) instead of burning. | This section measures your skin's tendency to produce melanin, a skin pigment that results in darker skin colour and causes dark spots, freckles, and dark-coloured scars. Melanin also helps the skin tan instead of burn. |
| 1 | After you have a pimple or ingrown hair, it’s followed by a dark brownish/black spot:   1. Never 2. Sometimes 3. Frequently 4. Always 5. I never get pimples or ingrown hairs | After you have acne or inflammation due to ingrown hairs that turn into brown/black spots:   1. Never 2. Sometimes 3. Often 4. Always 5. I've never had breakouts or any kind of inflammation | After you have acne or inflammation due to ingrown hair, do brown/dark spots appear?   1. Never 2. Sometimes 3. Often 4. Always 5. I never have acne or such inflammation. |
| 2 | After you cut yourself, how long does the brown (not pink) mark remain?   1. I don’t get a brown mark. 2. A week 3. A few weeks 4. Months | After a cut, how long does the brown (not pink) scar last?   1. I don't have a brown scar. 2. One week 3. few weeks 4. Months | How long do brown-coloured (not pink) scars last after being cut?   1. I don't have brown scars. 2. One week 3. Several weeks 4. Months |
| 3 | How many dark spots did you develop on your face when you were pregnant, on birth control pills, or taking hormone replacement therapy (HRT)?   1. None 2. One 3. A few 4. A lot 5. This question does not apply to me (because I am male, or because I have never been pregnant or taken birth control pills or HRT, or because I’m unsure whether I have dark spots). | How many dark spots appeared on your face when you were pregnant, on the contraceptive pill, or on hormone replacement therapy (HRT)?   1. There isn't any 2. One 3. A number of 4. Lots 5. This question doesn't apply (because I'm a man; or have never been pregnant, taken the birth control pill, or HRT; or because I'm not sure if I have dark spots). | How many dark spots appear on your face when you are pregnant, using contraception pills, or undergoing hormone replacement therapy (HRT)?   1. None 2. One 3. Several 4. Many 5. This question is not applicable (because I am a male, never been pregnant, used contraception pills, or HRT, or I am unsure if I have dark spots). |
| 4 | Do you have any dark spots or patches on your upper lip or cheeks? Or have you had any in the past that you’ve had removed?   1. No 2. I’m not sure. 3. Yes, they are (or were) slightly noticeable. 4. Yes, they are (or were) very noticeable. | Do you have any dark spots or stains on your upper lip or cheeks? Or have you ever had it and it's been removed?   1. No 2. I am not sure. 3. Yes, the spots/stains are not very clear. 4. Yes, those spots/stains are very obvious. | Do you have or have you ever had dark spots or freckles on your upper lip or cheek? Or have you had them removed?   1. No 2. I'm not sure. 3. Yes, the spots/freckles are not very prominent. 4. Yes, the spots/freckles are very prominent. |
| 5 | Do the dark spots on your face get worse when you go in the sun?   1. I have no dark spots. 2. Unsure 3. Slightly worse 4. A lot worse 5. I wear sunscreen on my face every day and never get sun. (Note: if you use constant sun protection because you’re afraid you might get dark patches or freckles, answer d.) | Are the dark spots on your face worse when exposed to the sun?   1. I don't have dark spots. 2. Not sure 3. Slightly worsened 4. Worse 5. I wear sunscreen on my face every day and never go out in the sun. (Note: if you always wear sun protection because you're afraid of dark spots or tan spots, answer d.) | Does the appearance of dark spots on your face worsen when exposed to the sun?   1. I don't have dark spots. 2. Not sure 3. Slightly worsen 4. Worsen 5. I wear sunscreen on my face every day and never expose it to sunlight. (Note: If you always wear sunscreen because you're afraid of dark spots or freckles, choose d.) |
| 6 | Have you been diagnosed with melasma, light or dark brown or gray patches, on your face?   1. No 2. Once, but it went away. 3. Yes 4. Yes, a severe case 5. Unsure | Have you ever been diagnosed with melasma, having light brown or dark brown or grayish patches, on your face?   1. No 2. One time, but it's gone. 3. Yes 4. Yes, including severe cases 5. Not sure | Have you ever been diagnosed with melasma, having light or dark brown or grayish patches on your face?   1. No 2. Once, but it has disappeared. 3. Yes 4. Yes, including severe cases 5. Not sure |
| 7 | Do you have, or have you ever had, small brown spots (freckles or sun spots) on your face, chest, back, or arms?   1. No 2. Yes, a few (one to five) 3. Yes, many (six to fifteen) 4. Yes, tons (sixteen or more) | Do you have, or have had, small brown spots (freckles or sunspots) on your face, chest, back, or arms?   1. No 2. Yes, several (one to five) 3. Yes, many (six to fifteen) 4. Yes, many (sixteen or more) | Do you have or have you ever had small brown spots (freckles or sunspots) on your face, chest, back, or arms?   1. No 2. Yes, a few (one to five) 3. Yes, many (six to fifteen) 4. Yes, a lot (sixteen or more) |
| 8 | When exposed to sun for the first time in several months, your skin:   1. Burns only 2. Burns then gets darker 3. Gets darker 4. My skin is already dark, so it is hard to see if it gets darker. (You can’t pick “I never had sun exposure.” Think of childhood experiences!) | When exposed to sunlight for the first time in months, your skin:   1. Just burned 2. The burn then gets darker 3. Get darker 4. My skin is already dark, so it's hard to tell if it's getting darker or not. (There's no "I've never been in the sun." Try to remember a childhood experience!) | When exposed to sunlight for the first time in several months, what happens to your skin?   1. Only burns 2. Burns and then tans 3. Tans 4. My skin is already dark, so it's hard to tell if it gets darker or not. (There's no option for "I have never been exposed to the sun." Try to remember your childhood experiences!) |
| 9 | What happens after you have had many days of consecutive sun exposure:   1. I sunburn and blister, but my skin does not change color. 2. My skin becomes slightly darker. 3. My skin becomes much darker. 4. My skin is already dark, so it is hard to see if it gets darker. 5. Unsure (Again, you can’t pick “I never had sun.” If you really have to pick e, first consider all childhood experiences.) | What happens after you've been exposed to the sun for a few consecutive days?   1. My skin is sunburned and blistered, but not discolored. 2. My skin has become a little darker. 3. My skin has become much darker. 4. My skin is already dark, so it's hard to tell if it's getting darker or not. 5. Not sure (again, there's no “I've never been in the sun.” If you do have to choose e, be sure to consider all of your childhood experiences.) | What happens after continuous exposure to sunlight for several days?   1. My skin gets sunburned and blisters, but does not change colour. 2. My skin gets slightly darker. 3. My skin gets significantly darker. 4. My skin is already dark, so it's hard to tell if it gets darker or not. 5. Not sure (again, there's no option for "I have never been exposed to the sun." If you really have to choose e, make sure you consider your childhood experiences.) |
| 10 | When you go in the sun, do you develop freckles (small 1–2 mm, pinpoint-sized flat spots)?   1. No, I never develop them. 2. I develop a few new small freckles each year. 3. I develop new freckles often. 4. My skin is already dark, so it is hard to see if I have freckles. 5. I never go in the sun. (Good for you!) | When exposed to sunlight, do you develop brown spots on your skin (which are flat to the surface of the skin, 1–2 mm in size like the tip of a needle)?   1. No, no such spots have ever appeared. 2. Every year a few new little spots appear. 3. New spots often appear 4. I have dark skin, so it's hard to see any brown spots. 5. I've never been under the sun. (Great!) | When exposed to sunlight, do brown spots (flat with the skin surface, measuring 1-2 mm like the tip of a needle) appear on your skin?   1. No, such spots never appear. 2. A few small spots appear every year. 3. New spots frequently appear. 4. My skin is already dark, so it's difficult to see if there are any brown spots. 5. I never expose myself to the sun. (Amazing!) |
| 11 | Did either of your parents have freckles? If so, please indicate how many. If neither or one parent did, respond to the question. If both did, answer the question as it relates to the parent with the most freckles.   1. No 2. A few on the face 3. Many on the face 4. Many on face, chest, neck, and shoulders 5. Unsure | Did your parents have brown spots? If yes, state how much. If neither parent has, or only one has brown spots, answer this question. If both have, choose the answer based on the parent with the most brown spots.   1. No 2. There are some on the face 3. There are many on the face 4. There are a lot of them on the face, chest, neck and shoulders 5. Not sure | Do your parents have brown spots? If yes, please indicate how many. If neither parent has them, or only one of them has brown spots, answer this question. If both parents have them, choose the answer based on the parent with the most brown spots.   1. No. 2. There are some on their faces. 3. There are many on their faces. 4. There are many on their faces, chest, neck, and shoulders. 5. Not sure. |
| 12 | What is your natural hair color? (If gray, state color before graying.)   1. Blond 2. Brown 3. Black 4. Red | What is your natural hair color? (If gray hair, state the color before gray hair appears.)   1. Blonde 2. Chocolate 3. Black 4. Red | What is your natural hair colour? (If greying, indicate the colour before the appearance of grey hair.)   1. Blonde. 2. Brown. 3. Black. 4. Red. |
| 13 | Do you have a history of melanoma yourself or in your immediate family?   1. No 2. One person in my family 3. More than one person in my family 4. I have a history of melanoma. 5. Unsure | Do you or your immediate family have a history of melanoma?   1. No 2. There is one person in my family 3. There is more than one person in my family 4. I have a history of melanoma. 5. Not sure | Do you or your immediate family have a history of melanoma?   1. No. 2. One person in my family. 3. More than one person in my family. 4. I have a history of melanoma. 5. Not sure. |
| 14 | If you have dark spots on your skin in areas of sun exposure, add 5 points to your score. | If you have dark spots on sun-exposed areas of your skin, add 5 points to your score. | If you have dark spots on sun-exposed areas of your skin, add 5 points to your score. |
|  | **Part Four**  **Wrinkled vs. Tight** | **Section Four**  **Wrinkles vs. Tight** | **Part Four**  **Wrinkles vs. Firmness** |
|  | This section measures your tendency to wrinkle, as well as how wrinkled you are right now. Some of my patients confessed that they cheated on this section to come out as a T—after I caught them doing it. Don’t do that! You’re only cheating yourself out of using preventative therapies that could prevent wrinkles. Changing your habits now could change your score in the future from a W to a T. So be honest and get the right treatments if you need them. | - This section measures your tendency to develop wrinkles, as well as how many wrinkles you currently have. - Some patients admit that they don't honestly answer this part as a T (Tight). Please don't do it! You are only doing yourself a disservice by depriving yourself of opportunities for therapies that prevent wrinkles. Changing your current habits can change your score in the future from Wrinkles to Tights. So, answer honestly and get the right treatment if you need it. | - This section assesses your propensity to develop wrinkles and the extent of wrinkles you presently have. - Some patients may not be completely truthful when responding to this section as "T" (Tight). Please refrain from doing so! By doing this, you only hinder yourself from receiving wrinkle-preventing therapy. Altering your current habits can potentially transform your score from "W" (Wrinkle) to "T" (Tight) in the future. Therefore, kindly provide honest answers and seek appropriate treatment if necessary.. |
| 1 | Do you have facial wrinkles?   1. No, not even with movement such as smiling, frowning, or lifting my eyebrows 2. Only when I move, such as smiling, frowning, or lifting my eye-brows 3. Yes, with movement and a few at rest without movement 4. Wrinkles are present even if I’m not smiling, frowning, or lifting my brows.   In answering questions 2–7, please respond according to how you would compare yourself and other family members to all other ethnic groups, not just your own. For family members who you may not have known, please ask other family members or refer to photographs, where possible. | Are there any wrinkles on your face?   1. No, not even during gestures like smiling, frowning, or raising an eyebrow 2. Only when moving your face, such as smiling, frowning, or raising your eyebrows 3. Yes, when moving the face, also slightly wrinkled when resting without movement 4. Wrinkles are there even if I don't smile, frown, or raise my eyebrows.   When answering questions 2-7, please answer by considering the comparison between you and your family members with other tribes in Indonesia. For family members you may not know, please ask other family members or see photos of them, if possible. | Do you have wrinkles on your face?   1. No, not even when making facial expressions like smiling, frowning, or raising eyebrows. 2. Only when making facial expressions like smiling, frowning, or raising eyebrows. 3. Yes, when making facial expressions and also some wrinkles when at rest without any movement. 4. I have wrinkles even when I'm not smiling, frowning, or raising eyebrows.   When responding to questions 2-7, kindly take into account the comparison between yourself and individuals belonging to other ethnicities in Indonesia. For family members whom you may not be acquainted with, please seek information from other family members or refer to their photographs, if available. |
| 2 | How old does/did your mother’s facial skin look?   1. Five to ten years younger than her age 2. Her age 3. Five years older than her age 4. More than five years older than her age 5. Not applicable; I was adopted or I cannot remember. | What age does your mother's facial skin look like?   1. Five to ten years younger than his age 2. Age-appropriate 3. Five years older than his age 4. More than five years older than his age 5. Not applicable; I was adopted or I don't remember. | At what age does your mother's facial skin appear?   1. Five to ten years younger than her actual age. 2. Same as her actual age. 3. Five years older than her actual age. 4. More than five years older than her actual age. 5. Not applicable; I am adopted or I don't remember. |
| 3 | How old does/did your father’s facial skin look?   1. Five to ten years younger than his age 2. His age 3. Five years older than his age 4. More than five years older than his age 5. Not applicable; I was adopted or I cannot remember. | How old does your father's facial skin look like?   1. Five to ten years younger than his age 2. Age-appropriate 3. Five years older than his age 4. More than five years older than his age 5. Not applicable; I was adopted or I don't remember. | At what age does your father's facial skin appear?   1. Five to ten years younger than his actual age. 2. Same as his actual age. 3. Five years older than his actual age. 4. More than five years older than his actual age. 5. Not applicable; I am adopted or I don't remember. |
| 4 | How old does/did your maternal grandmother’s facial skin look?   1. Five to ten years younger than her age 2. Her age 3. Five years older than her age 4. More than five years older than her age 5. Not applicable; I was adopted, never knew her, or cannot remember. | How old did your maternal grandmother's facial skin look like?   1. Five to ten years younger than his age 2. Age-appropriate 3. Five years older than his age 4. More than five years older than his age 5. Not applicable; I was adopted, never knew him, or don't remember. | At what age does your maternal grandmother's facial skin appear?   1. Five to ten years younger than her actual age. 2. Same as her actual age. 3. Five years older than her actual age. 4. More than five years older than her actual age. 5. Not applicable; I am adopted, never knew her, or don't remember. |
| 5 | How old does/did your maternal grandfather’s facial skin look?   1. Five to ten years younger than his age 2. His age 3. Five years older than his age 4. More than five years older than his age 5. Not applicable; I was adopted, never knew him, or cannot remember. | How old did your maternal grandfather's facial skin look like?   1. Five to ten years younger than his age 2. Age-appropriate 3. Five years older than his age 4. More than five years older than his age 5. Not applicable; I was adopted, never knew him, or can't remember him. | At what age does your maternal grandfather's facial skin appear?   1. Five to ten years younger than his actual age. 2. Same as his actual age. 3. Five years older than his actual age. 4. More than five years older than his actual age. 5. Not applicable; I am adopted, never knew him, or don't remember. |
| 6 | How old does/did your paternal grandmother’s facial skin look?   1. Five to ten years younger than her age 2. Her age 3. Five years older than her age 4. More than five years older than her age 5. Not applicable; I was adopted, never knew her, or cannot remember. | How old did your paternal grandmother's facial skin look like?   1. Five to ten years younger than his age 2. Age-appropriate 3. Five years older than his age 4. More than five years older than his age 5. Not applicable; I was adopted, never knew him, or don't remember. | At what age does your paternal grandmother's facial skin appear?   1. Five to ten years younger than her actual age. 2. Same as her actual age. 3. Five years older than her actual age. 4. More than five years older than her actual age. 5. Not applicable; I am adopted, never knew her, or don't remember. |
| 7 | How old does/did your paternal grandfather’s facial skin look?   1. Five to ten years younger than his age 2. His age 3. Five years older than his age 4. More than five years older than his age 5. Not applicable; I was adopted, never knew him, or cannot remember. | How old did your paternal grandfather's facial skin look like?   1. Five to ten years younger than his age 2. Age-appropriate 3. Five years older than his age 4. More than five years older than his age 5. Not applicable; I was adopted, never knew him, or can't remember him. | At what age does your paternal grandfather's facial skin appear?   1. Five to ten years younger than his actual age. 2. Same as his actual age. 3. Five years older than his actual age. 4. More than five years older than his actual age. 5. Not applicable; I am adopted, never knew him, or don't remember. |
| 8 | At any time in your life, have you ever tanned your skin on an ongoing basis for more than two weeks per year? If so, for how many total years did you do this? Please count tanning from playing tennis, fishing, playing golf, skiing, or other outdoor activities. The beach is not the only place you can get a tan.   1. Never 2. One to five years 3. Five to ten years 4. More than ten years | Throughout your age, have you ever tanned continuously for more than two weeks per year? If so, how many years did you do it in total? Also take into account the darkening (tanning) of the skin due to playing tennis, fishing, golf, skiing, or other outdoor activities. The beach isn't the only place that can make your skin darker (tan).   1. Never 2. One to five years 3. Five to ten years 4. More than ten years | Have you consistently exposed your skin to tanning for more than two weeks per year throughout your life? If so, for how many years in total? Take into account tanning resulting from activities such as tennis, fishing, golf, skiing, or other outdoor activities. It's important to note that beaches are not the sole locations where your skin can become tanned.   1. Never. 2. One to five years. 3. Five to ten years. 4. More than ten years. |
| 9 | At any time in your life, have you ever engaged in seasonal tanning of two weeks per year or less? (Yes, summer vacation counts!) If so, how often?   1. Never 2. One to five years 3. Five to ten years 4. More than ten years | Throughout your age, have you ever tanned due to the seasons, for two weeks or less a year? (Yes, including summer vacations!) If yes, how often?   1. Never 2. One to five years 3. Five to ten years 4. More than ten years | Have you engaged in seasonal tanning, exposing your skin for two weeks or less per year throughout your life? (Yes, this includes during summer vacations!) If so, how frequently did you do so?   1. Never. 2. One to five years. 3. Five to ten years. 4. More than ten years. |
| 10 | Based on the places you’ve lived, how much daily sun exposure have you received in your life?   1. Little; I’ve mostly live at places that are gray and overcast. 2. Some; I’ve lived in less sunny climes at times, but also in places with more regular sun. 3. Moderate; I’ve lived in places with a fair amount of sun exposure. 4. A lot; I’ve lived in tropical, Southern, or very sunny locales. | Based on the places you have lived, how much daily sun exposure did you get in your entire life?   1. Not enough; i live mostly in shady and cloudy places. 2. A little; I've lived in places that didn't get much sun, but I've also lived in places with plenty of sun 3. Enough; i live in places with a fair amount of sun exposure 4. Lots; i live in the tropics or places with hot sun. | Based on the places you have lived, how much daily sun exposure have you had throughout your life?   1. Low; I have mostly lived in shaded and cloudy areas. 2. Moderate; I have lived in places with some sun exposure but not excessively. 3. High; I have lived in places with significant sun exposure. 4. Very high; I have lived in tropical regions or places with intense sunlight. |
| 11 | How old do you think you look?   1. One to five years younger than your age 2. Your age 3. Five years older than your age 4. More than five years older than your age | How old do you think you look?   1. One to five years younger than your actual age 2. Same as your age 3. Five years older than your age 4. More than five years older than your age | In your opinion, how old do you appear?   1. One to five years younger than your actual age. 2. Same as your actual age. 3. Five years older than your actual age. 4. More than five years older than your actual age. |
| 12 | During the last five years, how often have you allowed your skin to tan either intentionally or unintentionally through outdoor sports or other activities?   1. Never 2. Once a month 3. Once a week 4. Daily | Over the past five years, how often have you allowed your skin to darken intentionally or unintentionally by playing sports or other outdoor activities?   1. Never 2. Once a month 3. Once a week 4. Every day | In the past five years, how often have you unintentionally or intentionally tanned your skin through sports or outdoor activities?   1. Never. 2. Once a month. 3. Once a week. 4. Every day. |
| 13 | How often, if ever, have you been to a tanning bed?   1. Never 2. One to five times 3. Five to ten times 4. Many times | How often, if ever, do you use tanning beds?   1. Never 2. One to five times 3. Five to ten times 4. Very often | How often, if ever, do you use a tanning bed?   1. Never. 2. One to five times. 3. Five to ten times. 4. Very often. |
| 14 | Over your entire life, how many cigarettes have you smoked (or been exposed to)?   1. None 2. A few packs 3. Several to many packs 4. I smoke every day. 5. I’ve never smoked but I’ve lived with, been raised by, or worked with people who regularly smoked in my presence. | In your lifetime, how much have you smoked (or been exposed to) cigarettes?   1. Never 2. Several packs 3. Until packed 4. I smoke every day. 5. I have never smoked but have lived, been raised by, or worked with people who smoke frequently around me. | Throughout your life, how much have you smoked (or been exposed to smoke)?   1. Never. 2. A few packs. 3. Several packs. 4. I smoke every day. 5. I have never smoked but have lived, been raised by, or worked with people who smoke around me frequently. |
| 15 | Please describe the air pollution where you reside:   1. The air is fresh and clean. 2. For part of the year, but not all of the year, I reside in a place with clean air. 3. The air is slightly polluted. 4. The air is very polluted. | Please describe the air pollution where you live:   1. Fresh and clean air. 2. I live in a place with clean air for some time, but not a full year. 3. The air is slightly polluted. 4. The air is very polluted. | Please describe the air pollution in your place of residence:   1. Fresh and clean air. 2. I have lived in a place with clean air for some time but not a full year. 3. Slightly polluted air. 4. Highly polluted air. |
| 16 | Please describe the length of time that you have used retinoid facial creams such as retinol, Renova, Retin-A, Tazorac, Differin, or Avage:   1. Many years 2. Occasionally 3. Once for acne when I was younger 4. Never | How long have you been using face creams that contain retinoids such as retinol, Renova, Retin-A, Tazorac, Differin, or Avage?   1. Years 2. Occasionally 3. Once for acne when I was young 4. Never | How long have you been using face creams containing retinoids like retinol, Renova, Retin-A, Tazorac, Differin, or Avage?   1. Several years. 2. Occasionally. 3. Once for acne when I was younger. 4. Never. |
| 17 | How often do you currently eat fruits and vegetables?   1. At every meal 2. Once a day 3. Occasionally 4. Never | How often do you eat fruits and vegetables?   1. Every meal 2. Once a day 3. Sometimes 4. Never | How often do you eat fruits and vegetables?   1. Every meal. 2. Once a day. 3. Occasionally. 4. Never. |
| 18 | Over your lifetime, what percentage of your daily diet has consisted of fruits and vegetables? (Note: don’t count juices unless they are freshly squeezed.)   1. 75–100 percent 2. 25–75 percent 3. 10–25 percent 4. 0–10 percent | Throughout your life, what percentage of your daily diet consisted of fruits and vegetables? (Note: juices are not included, unless freshly squeezed.)   1. 75 – 100 percent 2 2. 5 – 75 percent 3. 10 – 25 percent 4. 0 – 10 percent | Throughout your life, what percentage of your daily diet consists of fruits and vegetables? (Note: Exclude juices, except freshly squeezed ones.)   1. 75 - 100 percent. 2. 25 - 75 percent. 3. 10 - 25 percent. 4. 0 - 10 percent. |
| 19 | What is your natural skin color (without tanning or self-tanners)?   1. Dark 2. Medium 3. Light 4. Very light | What is your natural skin tone (without sun tanning or tanning products)?   1. Dark 2. Olive or tan 3. Bright/white 4. Very bright/very white | What is your natural skin colour (without sun exposure or tanning products)?   1. Dark. 2. Olive or tan. 3. Fair/white. 4. Very fair/very white. |
| 20 | What is your ethnicity? (Please choose best answer.)   1. African/African-American/Aboriginal/Maori/Caribbean/Black 2. Asian/Indian/Mediterranean/Other 3. Latin-American/Hispanic/Mideastern 4. Caucasian | What is your ethnicity? (Please choose the best answer.)   1. African/African-American/Aboriginal/Maori/Caribbean/Black. 2. Asian/Indian/Mediterranean/Other. 3. Latin American/Hispanic/Middle Eastern. 4. Caucasian. | What is your ethnicity? (Please choose the best answer.)   1. African/African-American/Aboriginal/Maori/Caribbean/Black. 2. Asian/Indian/Mediterranean/Other. 3. Latin American/Hispanic/Middle Eastern. 4. Caucasian. |
| 21 | If you are sixty-five years or older, add 5 points to your score. | If you are 65 or older, add 5 points to your score. | If you are 65 years or older, add 5 points to your score. |
