# Supplementary material for: Validity, reliability, and comparison of the Indonesian version of two baumann skin type indicator (BSTI) questionnaires
Source: PLoS One. 2026 Apr 2;21(4):e0343028. doi: 10.1371/journal.pone.0343028 (PMC13046154; doi:10.1371/journal.pone.0343028)
Supplement: S2 Table — (DOCX) [file pone.0343028.s002.docx]

**S2 Table. Back translation of the 2022 version of BSTI**

| No | Baumann Skin Type Indicator Assessment  for Research Purposes Only  **(Original)** | Baumann Skin Type Indicator Assessment For Research Purposes Only  **(Back Translation 1)** | Baumann Skin Type Indicator Assessment For Research Purposes Only  **(Back Translation 2)** |
| --- | --- | --- | --- |
|  | The following diagnostic questionnaire will determine the Baumann Skin Type® of 16 possible  combinations made up of the 4 barriers to skin health:   - Skin Dehydration: Dry (D) or Oily (O) - Skin Sensitivity: Sensitive (S) or Resistant ® - Skin Pigmentation: Pigmented (P) or Non-Pigmented (N) - Skin Aging: Wrinkle Prone (W) or Tight (T)   *Instructions are italicized.* Red text is for scoring purposes, | The following diagnostic questionnaire will determine the Baumann® Skin Type out of 16 possible combinations consisting of 4 parameters for skin health:   - Dehydrated skin: Dry (D) or Oily (O) - Skin sensitivity: Sensitive (S) or Resistant (R) - Skin Pigmentation: Pigmented (P) or Non-pigmented (N) - Skin aging: Wrinkle Prone (W) or Tight (T)   *Instructions in italics.* Red text is for grading purposes. | The following diagnostic questionnaire will determine the Baumann® Skin Type out of 16 possible combinations consisting of 4 parameters for healthy of skin:   - Dehydrated skin: Dry (D) or Oily (O) - Skin sensitivity: Sensitive (S) or Resistant (R) - Skin Pigmentation: Pigmented (P) or Non-pigmented (N) - Skin Aging: Wrinkle Prone (W) or Tight (T)   *Instructions are in italics.* Red texts are for assessment purposes. |
| 1 | **Personal Information**  What is your gender?   - Male - Female - Prefer not to say   *If “Female” or “Prefer not to say” present the following question:*  1A) *Are you pregnant or breastfeeding?*   - Yes - No | **Personal information**  What is your gender?   - Male - Female - Prefer not to mention   *If “Female” or “Prefer not to mention” continue with the following questions: 1A) Are you pregnant or breastfeeding?*   - Yes - No | **Personal information**  What is your gender?   - Male - Female - Prefer not to specify   *If your answer is “Female” or “Prefer not to specify” continue with the following questions:*  *1A) Are you pregnant or breastfeeding?*   - Yes - No |
| 2 | What is your birth month and year?  *Calculate Current Age. 30 years old or above are automatically designed as* ***Wrinkle Prone (W)***  **Barrier 1**: Skin Dehydration (**D**ry or **O**ily) | What month and year were you born?  *Calculate Current Age. Age 30 years or more is automatically called* ***Vulnerable Wrinkles/Wrinkle Prone (W)***  **Parameter 1:** Dehydrated skin (Dry **[D]** or Oily **[O]**) | What month and year were you born?  *Calculate your age at the moment. If you are 30 years old or more, then you are classified as* ***Vulnerable Wrinkles/Wrinkle Prone (W)***  **Parameter 1:** Dehydrated skin (Dry **[D]** or Oily **[O]**) |
| 3 | Please check all that are true about your facial skin. (Multiple answers are preferred.)   - I can use any soap to wash my face without developing dryness = +2 - I do not apply any products to my facial skin after cleansing = +1 - I never or only occasionally apply a moisturizer = +2 - I apply a moisturizer to my face once a day = –1 - I apply a moisturizer to my face twice a day = –2 | Check each statement that is true about your facial skin. (More than one is recommended.)   - I can use any soap to wash my face without dry sensation = +2 - I don't use any products on my skin after cleansing = +1 - I never or only occasionally use moisturizer = +2 - I use facial moisturizer once a day = –1 - I use facial moisturizer twice a day = –2 | Check the following statements, choose those most true about your facial skin. (It is recommended to choose more than one.)   - I can use any soap to wash my face without dry sensation = +2 - I don't use any products on my skin after cleansing = +1 - I never or only occasionally use moisturizer = +2 - I use facial moisturizer once a day = –1 - I use facial moisturizer twice a day = –2 |
| 4 | Please check all that are true about your facial skin. (Multiple answers are preferred.)   - My facial skin is rough or dry = –2 - My facial skin is oily in some areas = +2 - My face is very oily = +3 - My face is uncomfortable if I do not use a moisturizer = –2 - I like the feel of heavy creams and/or oil on my skin = –3 - None of the above = 0   *Sum score for Questions 3 & 4 to achieve the Oily or Dry Score*  *Determine Skin Dehydration Designation:*   - –10 to 0 = **Dry (D)** - +1 to +10 = **Oily (O)**   *Determine Skin Dehydration Sub-Type Designation:*   - –10 to –7 = “Very Dry” = **D2** - –6 to –2 = “Dry” = **D1** - –1 to +1 = ‘Combination” = **D** - +2 to +6 = “Oily” = **O1** - +7 to +10 = “Very Oily” = **O2**   **Barrier 2:** Skin Sensitivity (**S**ensitive or **R**esistant) | Put a tick mark on each statement that is true about your facial skin. (More than one is recommended.)   - My facial skin is rough or dry = -2 - My facial skin is oily in some areas = +2 - My face is very oily = +3 - My facial skin feels uncomfortable without using moisturizer = -2 - I like the feel of heavy cream and/or oil on my skin = -3 - None of the above = 0   *Add up the points in Questions 3 & 4 to get an Oily (O) or Dry (D) Score*  *Define Dehydrated Skin Category:*   - –10 hingga 0 = **Dry (D)** - +1 hingga +10 = **Oily(O)**   *Define Skin Dehydration Sub-Type Category:*   - –10 to –7 = “Very Dry” = **D2** - –6 to –2 = “Dry” = **D1** - –1 to +1 = “Combination” = **D** - +2 to +6 = “Oily” = **O1** - +7 to +10 = “Very Oily” = **O2**   **Parameter 2:** Skin Sensitivity (**S**ensitive or **R**esistant) | Put a tick mark on any statement that is true about your facial skin. (It is recommended to choose more than one.)   - My facial skin is rough or dry = -2 - My facial skin is oily in some areas = +2 - My face skin is very oily = +3 - My facial skin feels uncomfortable without using moisturizer = -2 - I feel like heavy cream and/or oil applied on my skin = -3 - None of the above = 0   *Add up the points in Questions 3 & 4 to determine whether your skin is Oily (O) or Dry (D)*  *Define yours according to the following Skin Dehydration Category:*   - 10 sampai 0 = **Dry(D)** - +1 sampai +10 =**Oily(O)**   Define your according to the following Skin Dehydration Sub-Type Category:   - –10 to –7 = “Very Dry” = D2 - –6 to –2 = “Dry” = D1 - –1 to +1 = “Combination” = D - +2 to +6 = “Oily” = O1 - +7 to +10 = “Very Oily” = O2   **Parameter 2:** Skin Sensitivity (Sensitive or Resistant) |
| 5 | Check all the following that you are prone to: (Multiple answers allowed)   - Acne (pimples) = S - Facial redness and or flushing = S - Stinging or burning of skin = S - Allergic reactions to skin care products = S (*If they select this present question 5.1*) - Irritation from shaving the face (ask for Males only) = S - None of the above = R   *Determine Skin Sensitivity Designation:*   - If at least one “S” choice was selected, designation is **Sensitive (S)** - If None of the above, designation is **Resistant (R)**   *Determine Skin Sensitivity Sub-Type Designation: (combine for multiple selections e.g., S1S3)*   - Acne (pimples) = **S1** - Facial redness and or flushing = **S2** - Stinging or burning of skin = **S3** - Allergic reactions to skin care products = **S4** - Irritation from shaving the face = **S5**   *This question does not contribute to scoring – informational purposes only*  5.1) What are you allergic to that touches your skin? (Multiple answers allowed)   - I do not know - Adhesive tape - Balsam of Peru - Cetyl alcohol - Cobalt - Color cosmetics such as eyeshadow - Daisy family of plants: [Ragweed, Arnica, Calendula (Marigolds), Chamomile, Chrysanthemums, Echinacea, Feverfew] - Formaldehyde - Fragrance and Perfume - Gold - Hair Dyes, Latex (gloves) - Lidocaine (topical and injectable anesthesia) - Mascara - Nail polish - Neomycin or bacitracin (topical antibiotic) - Nickel (Jewelry) - Paraben - Preservatives - Quaternium- 15 - Silver - Sunscreen - Thimerosal - Toothpaste - Triclosan - Underarm deodorant - What I am allergic to is not on this list. Please specify:   **Barrier 3:** Skin Pigmentation (**P**igmented or **N**on-Pigmented) | Put a check mark on the condition that you are prone to experiencing: (More than one answer may be allowed)   - Acne (acne) = S - Facial redness and/or flushing = S - Stinging or burning sensation on the skin = S - Allergic reaction to skin care products = S (If you choose this, go to question 5.1) - Irritation from shaving the face (men only) = S - None of the above = R   *Define Skin Sensitivity Category:*   - If you select at least one “S” answer, it is categorized as Sensitive (S) - If none of the above, it is categorized as Resistance (R)   *Determine Skin Sensitivity Subtype Category: (combine if more than one answer, for example S1S3)*   - Acne (acne) = **S1** - Facial redness and/or flushing = **S2** - Stinging or burning sensation on the skin = **S3** - Allergic reaction to skin care products = **S4** - Irritation from shaving = **S5**   *This question is not included in the assessment - for informational purposes only 5.1) Which ones cause allergies if they touch your skin? (Answers may be more than one)*   - I don't know - Masking tape - Peruvian balsam - Cetyl alcohol - Cobalt - Colored cosmetics such as eye shadow - Plants of the Daisy family: [Ragweed, Arnica, Calendula (Marigolds), Chamomile, Chrysanthemum, Echinacea, Feverfew] - Formaldehyde - Fragrances and Perfumes - Gold - Hair dye, latex (gloves) - Lidocaine (topical and injectable anesthetics) - Mascara - Nail polish - Neomycin or Bacitracin (topical antibiotics) - Nickel (Jewelry) - Parabens - Preservatives - Quaternium- 15 - Silver - Sunscreen - Thimerosal - Toothpaste - Triclosan - Underarm deodorant - What I'm allergic to is not on this list. Please mention:   **Parameter 3:** Skin Pigmentation (Pigmented or Non-Pigmented) | Put a check mark on the condition that you are prone to: (You are allowed to provide more than one answer)   - Acne = S - Facial redness and/or flushing = S - Stinging or burning sensation on the skin = S - Allergic reaction to skin care products = S (If you choose this, go to question 5.1) - Irritation from shaving facial hairs (men only) = S - None of the above = R   *Define your Skin Sensitivity Category:*   - If you select at least one “S” answer, your skin is categorized as Sensitive (S) - If you did not select any “S” answer, your skin is categorized as Resistance (R)   Determine your skin Sensitivity Sub-Type Category: (combine if your answer are more than one, for example S1S3)   - Acne = **S1** - Facial redness and/or flushing = **S2** - Stinging or burning sensation on the skin = **S3** - Allergic reaction to skin care products = **S4** - Irritation from shaving facial hairs = **S5**   This question is not included in the assessment - for informational purposes only  5.1) Which ones of the following that cause allergies when contacting with your skin? (You are allowed to provide more than one answer)   - I don't know - Masking tape - Peruvian balsam - Cetyl alcohol - Cobalt - Coloured cosmetics such as eye shadow - Plants of the Daisy family: [Ragweed, Arnica, Calendula (Marigolds), Chamomile, Chrysanthemum, Echinacea, Feverfew] - Formaldehyde - Fragrances and Perfumes - Gold - Hair dye, latex (gloves) - Lidocaine (topical and injectable anesthetics) - Mascara - Nail polish - Neomycin or Bacitracin (topical antibiotics) - Nickel (Jewellery) - Parabens - Preservatives - Quaternium-15 - Silver - Sunscreen - Thimerosal - Toothpaste - Triclosan - Underarm deodorant - What I'm allergic to is not on this list. Please specify:   **Parameter 3:** Skin Pigmentation (Pigmented or Non-Pigmented) |
| 6 | Do you have uneven skin pigmentation and want to lighten dark spots and patches on your face as seen in the pictures? (Choose one)  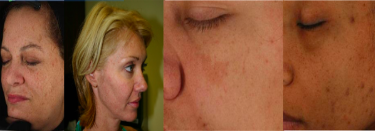   - No, I have no uneven skin pigmentation on my face = **N** - Yes, I have uneven skin pigmentation and I want to lighten the dark, uneven spots on my face = **P** - Yes, I have freckles or uneven skin tone, but I do not want to lighten the spots on my face = **N**   If choice is selected, then add this end text:  “You have uneven skin tone but have chosen not to treat the brown spots at this time. For this reason, you have been skin typed as a “Non-Pigmented Type”.  Based on selection, Designation is **Non-Pigmented (N) or Pigmented (P)**  **Barrier 4:** Skin Aging (**W**rinkle Prone or **T**ight) | Do you have uneven skin pigmentation and want to lighten dark spots and blotches on your face as shown in the picture? (Select one)  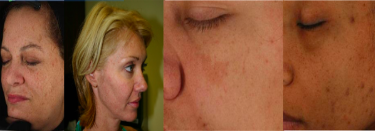   - No, I don't have uneven skin pigmentation on my face = N - Yes, I have uneven skin pigmentation and I want to lighten dark and uneven spots on my face =P - Yes, I have freckles or uneven skin tone, but I don't want to lighten the spots on my face = N   If you choose this answer, then add the following notes: “You have an uneven skin tone but prefer not to treat those brown spots at this point. Therefore, your skin is categorized as “Non-Pigmented Type.”  Based on the answer choices, categorize as **Non-pigmented (N) or Pigmented (P)**  **Barrier 4:** Aging Skin (Prone to Wrinkles or Tightness) | Do you have uneven skin pigmentation and want to lighten dark spots and blotches on your face as shown in the picture? (Select one)  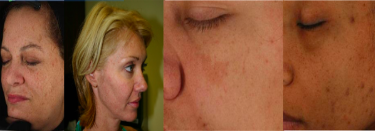   - No, I don't have uneven skin pigmentation on my face = N - Yes, I have uneven skin pigmentation and I want to lighten the freckles and uneven spots on my face =P - Yes, I have freckles or uneven skin tone, but I don't want to lighten them = N   If you choose this answer, then add the following notes: “You have an uneven skin tone but prefer not to treat those freckles at this point. Therefore, your skin is categorized as “Non-Pigmented Type.”  Based on the answers, determine skin pigmentation category as **Non-pigmented (N)** or, otherwise **Pigmented (P)**  **Barrier 4:** Skin Aging (Prone to Wrinkles or Tight) |
| 7 | Check all that apply to you. (Multiple answers allowed)   1. I have smoked over 50 cigarettes or cigars in my life. 2. I am exposed to secondhand smoke on a weekly basis 3. I currently smoke cigarettes or cigars 4. I have been to a tanning bed more than 3 times in my life 5. I am exposed to sun over 3 hours a week 6. I spend over 3 hours in a week close to a window during daylight (includes driving) 7. My face has been sunburned and peeled more than twice in my life 8. I do not take daily antioxidant supplements like Vitamin E and C. 9. I often get less than 7 hours of sleep a night 10. I feel stress at least 2 hours a day 11. One of my parents has more wrinkles than others their age. 12. I eat sugary foods over 3 times a week. 13. I exercise less than 3 hours a week 14. I do not eat fruit or vegetables every day 15. I do not wear sunscreen every day 16. I do not wear sunscreen during outdoor activities 17. None of the above   If 3 or more are selected below (except choice “None of the above”) designation is **Wrinkle Prone (W)**. Also, if age is 30 years or more designation is **Wrinkle-Prone (W).** All others are **Tight (T).**  Scoring Example below:  D/O Score = -8 = D= Very Dry  S/R Score and sensitivity subtype = S1= Acne, S2= Redness/ Rosaces, S3= Stinging, S4= Dermatitis  Type (Susceptible to Allergens and Irritants)  P/N Score = P  W/T Score = W  Overall Result = DSPW  Detailed Result = D2S1S2PW= Very dry, acne, rosacea, uneven skin pigment and wrinkle prone. | Put a check mark on the statement that suits you. (Answers may be more than one)   1. I have smoked more than 50 cigarettes or cigars in my life. 2. I am passively exposed to secondhand smoke every week 3. I currently smoke or smoke cigars 4. Throughout my life, I have used tanning beds more than 3 times 5. I am exposed to the sun more than 3 hours a week 6. I am near a window for more than 3 hours during the day (including driving) 7. Throughout my life, my face has been sunburned and peeled more than twice 8. I do not take daily antioxidant supplements such as Vitamins E and C. 9. I often sleep less than 7 hours per night 10. I feel stressed for at least 2 hours a day 11. One of my parents has more wrinkles than most of his peers. 12. I eat sweets more than 3 times a week. 13. I exercise less than 3 hours a week 14. I eat fruit and vegetables not every day 15. I don't wear sunscreen every day 16. I do not wear sunscreen during outdoor activities 17. None of the above   If you choose 3 or more answers (not including the option "None of the above") are categorized as **Wrinkle Prone (W)**. If you are 30 years old or older, you are also categorized as **Wrinkle Prone (W).** Apart from those described, it is categorized as **Tight (T).**  Below is an example of an assessment:  D/O Score = -8 = D = Very Dry  S/R score and sensitivity subtype = S1 = Acne, S2 = Redness/Rosasea, S3 = Stinging, S4 = Type of Dermatitis (Susceptible to Allergens and Irritants)  P/N score = P  W/T score = W  General Results = DSPW  Detailed Results = D2S1S2PW= Very dry, acne, rosacea, uneven skin pigmentation and prone to wrinkles. | Put a check mark on the statement that best suits you. (You are allowed to provide more than one answer)   1. I have smoked more than 50 cigarettes or cigars in my life. 2. I am passively exposed to cigarettes smoke weekly 3. I currently smoke cigars 4. Throughout my life, I have used tanning beds more than 3 times 5. I am exposed to the sun more than 3 hours a week 6. I am near a window for more than 3 hours during the day (including driving) 7. Throughout my life, my face has been sunburned and peeled more than twice 8. I do not take daily antioxidant supplements such as Vitamins E and C. 9. I often sleep less than 7 hours per night 10. I feel stressed for at least 2 hours a day 11. One of my parents has more wrinkles than most of his peers. 12. I eat sweets more than 3 times a week. 13. I exercise less than 3 hours a week 14. I don’t eat fruit and vegetables every day 15. I don't wear sunscreen every day 16. I don’t wear sunscreen during outdoor activities 17. None of the above   If you provide 3 answers or more (not including the option “None of the above”), then your skin is categorized as Wrinkle Prone (W). If you are 30 years old or older, you are also categorized as Wrinkle Prone (W). Apart from those described, it is categorized as Tight (T).  See the following for an example of assessment:  D/O Score = -8 = D = Very Dry  S/R score and sensitivity subtype = S1 = Acne, S2 = Redness/Rosacea, S3 = Stinging, S4 = Type of Dermatitis (Susceptible to Allergens and Irritants) P/N score = P  W/T score = W  General Results = DSPW  Detailed Results = D2S1S2PW= Very dry, acne, rosacea, uneven skin pigmentation and prone to wrinkles. |
